# A randomised controlled trial evaluating the effect of an individual auditory cueing device on freezing and gait speed in people with Parkinson's disease

**DOI:** 10.1186/1471-2377-8-46

**Published:** 2008-12-11

**Authors:** Sean Ledger, Rose Galvin, Deirdre Lynch, Emma K Stokes

**Affiliations:** 1 Department of Physiotherapy, School of Medicine, Trinity College Dublin, Dublin, Republic of Ireland

## Abstract

**Background:**

Parkinson's disease is a progressive neurological disorder resulting from a degeneration of dopamine producing cells in the substantia nigra. Clinical symptoms typically affect gait pattern and motor performance. Evidence suggests that the use of individual auditory cueing devices may be used effectively for the management of gait and freezing in people with Parkinson's disease. The primary aim of the randomised controlled trial is to evaluate the effect of an individual auditory cueing device on freezing and gait speed in people with Parkinson's disease.

**Methods:**

A prospective multi-centre randomised cross over design trial will be conducted. Forty-seven subjects will be randomised into either Group A or Group B, each with a control and intervention phase. Baseline measurements will be recorded using the Freezing of Gait Questionnaire as the primary outcome measure and 3 secondary outcome measures, the 10 m Walk Test, Timed "Up & Go" Test and the Modified Falls Efficacy Scale. Assessments are taken 3-times over a 3-week period. A follow-up assessment will be completed after three months. A secondary aim of the study is to evaluate the impact of such a device on the quality of life of people with Parkinson's disease using a qualitative methodology.

**Conclusion:**

The Apple iPod-Shuffle™ and similar devices provide a cost effective and an innovative platform for integration of individual auditory cueing devices into clinical, social and home environments and are shown to have immediate effect on gait, with improvements in walking speed, stride length and freezing. It is evident that individual auditory cueing devices are of benefit to people with Parkinson's disease and the aim of this randomised controlled trial is to maximise the benefits by allowing the individual to use devices in both a clinical and social setting, with minimal disruption to their daily routine.

**Trial registration:**

The protocol for this study is registered with the US NIH Clinical Trials Registry (NCT00727467).

## Background

Parkinson's disease (PD) is a progressive neurological disorder resulting from a degeneration of dopamine producing cells in the substantia nigra [1]. Clinical symptoms such as tremor, rigidity, bradykinesia, hypokinesia and postural instability classically affect gait pattern and motor performance [2,3]. Typically, people who present with PD have an inability to maintain internal gait rhythm [4], resulting in an abnormal gait pattern including short shuffling steps, decreased arm-swing [5], freezing [4-6], festination [7], decreased stride length [8,9], reduced walking speed and increased cadence and double stance time [2,4].

Rubenstein and colleagues [10] advocate the use of cueing devices in physiotherapy, as such devices have demonstrated an immediate effect on gait, with improvements in walking speed, stride length and cadence [8,9]. Cueing is defined as using external temporal or spatial stimuli to facilitate movement, gait initiation and continuation [6], and may be used effectively in a home environment for the management of gait and freezing in individuals with PD [11]. The RESCUE trial [11] demonstrated the efficacy of the use of individual auditory cueing devices (IACD). However, the technology used in this protocol is not commercially available to date. Therefore, in this article we describe a protocol to evaluate the effect of a widely available IACD – the Apple iPod-shuffle™ (iPod) on freezing and gait speed.

The iPod is an off-the-shelf, low cost MP3 player, combining the latest auditory technology with an aesthetically appealing, built-in clip design and user-friendly interface. The iPod can be attached to any item of clothing without being conspicuous and the in-ear headphones have excellent sound quality and fit comfortably and securely in the ear.

It is not exactly clear how AC improves gait, however McIntosh and colleagues [12] hypothesise that an external AC compensates for the defective internal rhythm of the basal ganglia. Research also suggests that AC may provide a rhythmic training mechanism because the effects of AC on the individual remained even after cues were removed [11-14]. In the study by McIntosh and colleagues [12], people with PD who listened to music with an overlaid AC whilst exercising showed lasting gait improvements, compared to those who exercised without an AC. In a similar study [14], individuals with PD who listened to music with an AC on a daily basis for a month without gait training showed significant improvements in gait velocity and step length.

### Background Work

A systematic review of randomised control trials (RCTs) was explored [15]. The review demonstrated that significant improvements in walking speed, stride length and cadence were evident in the 'experimental' groups when AC was used as an intervention [16,17]. The primary gait deficiency in people with PD has been described as an inability to generate sufficient amplitude of movement [18]; therefore cues should be aimed at increasing step amplitude [18] and enlarging stride length to have a maximum impact on normalising gait patterns of people with PD [19].

The review [15] also suggested that by increasing the auditory cueing (AC) frequency by +10% to 20% of individuals with PD natural step frequency, there is an immediate effect on walking speed and stride length [19-21]. However, the effects of increased stride length have been reported as inconsistent in people with PD who freeze [22]. Preliminary findings on people with PD who freeze indicate that lowering the AC frequency by 10% (-10%) of the individuals self-selected walking speed can help to improve their stride length [22,23]. Therefore the investigators calculated and pre-loaded 100 baseline frequencies (less 10%) in the sound of a metronome beat in an MP3 format (30 – 130 BPM) onto a DVD-R [24].

A user manual was created with a set of instructions that gives each physiotherapist a step-by-step guide on how to calculate the correct beat for their participants and how to successfully upload this beat onto the iPod. In addition physiotherapists in participating centres attended a training session on how to complete the tasks. A pack including the DVD-R, user manual, information leaflet and consent form was sent to participating centres. Recruitment is due to commence in January 2009.

In addition, other authors have found that a single task attention strategy such as walking in a straight line, with a single auditory cue, resulted in significant normalisation of gait parameters with improvements in walking speed, step amplitude, and step frequency [20,23,25-30]. The results of a recent study demonstrated a significant increase of 0.7 m in mean step length with AC compared to walking with no cues in a dual motor task, and significant increases in step amplitude with rhythmic auditory cueing during dual and multi-task performances in the home [23].

Using this body of evidence, the investigators developed a protocol to evaluate the impact of a low cost, widely available individual auditory cueing device on freezing and gait speed in people with Parkinson's disease.

### Aims and Objectives of the Study

The primary aim of the study is to evaluate the effect of an IACD i.e. Apple iPod-shuffle™, on freezing and gait speed in people with a diagnosis of PD through the implementation of a randomised controlled trial. A secondary aim of the study is to evaluate the impact of such a device on the quality of life of people with PD through the administration of a self-report questionnaire. The IACD will be pre-loaded with an individualised auditory cueing frequency (metronome sound) matched to the walking speed of the person.

## Methods

### Study Design

A randomised cross over design will be used. Forty-seven subjects will be randomised into either Group A or Group B using sealed, computer generated random numbers.

### Ethical Considerations

The study has obtained ethical approval from Trinity College, University of Dublin Research Ethics Committee (3/7/08).

### Intervention

On Days 1–8 of the trial, the participants in Group A and Group B will be given the iPod, loaded with podcasts. During this period they will familiarise themselves with the functionality of the device i.e. turning the device on and off, increasing and decreasing the volume and inserting the earphones into the ear. They will be instructed to use the device only when sitting at home, and also instructed that the device should not be turned on when walking or performing any mobility related or daily tasks. Investigators will schedule a phone call to check the device is being used properly and that the participant is not having any problems.

On Days 8–15, participants in Group A will be allocated to the 'intervention' phase. Each participant will be given an iPod containing an auditory cue in the form of a continuous metronome beat, individualised to the person's walking frequency (less 10%). Participants will be instructed to listen to the cueing when they are performing any mobility related tasks. They will be encouraged to listen to the rhythmical cue and to try to match their heel strike with the beat of the metronome sound on the device. During this time period, participants in Group B will be allocated to the 'control' phase. Participants will be provided with the iPod shuffle containing no music or metronome beat. Participants will be instructed to continue using the device in a similar manner to that of the first week. On Days 15–23, participants in Group A will be allocated to the 'control' phase and participants in Group B will be allocated to the 'intervention' phase. The progression from screening and enrollment to randomisation is illustrated in Figure legend 1.

**Figure 1:**
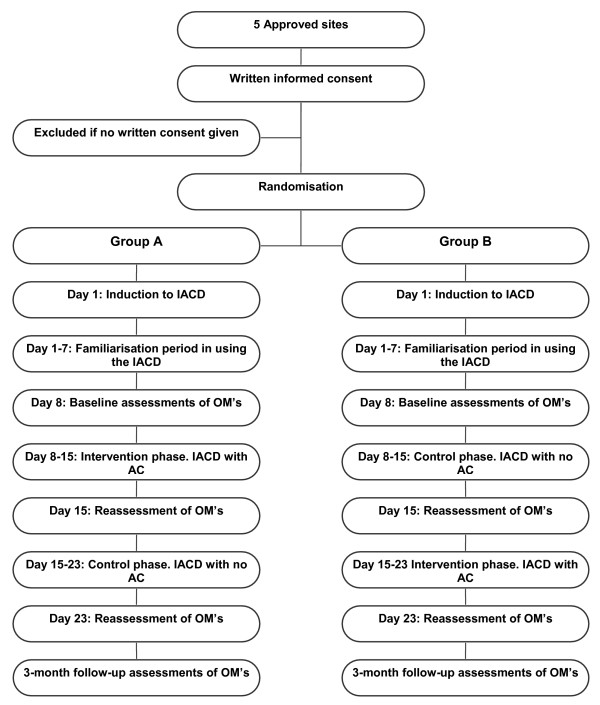
Outline of RCT study design.

### Outcome Measures

Assessments will be taken 3 times over the 3-week period on days 8, 15 and 23. A follow-up assessment will be completed after 3 months. The primary SOM in this study is the Freezing of Gait Questionnaire (FOGQ) [31]. The severity of freezing of gait is most accurately assessed by the FOGQ. The questionnaire focuses solely on freezing and does not include items relating to falling or festination. The FOGQ has been shown to be both valid and reliable as an outcome measure in people with PD [31].

Secondary outcomes will also be utilised including the 10MWT [32], TUG [33] and the MFES [34]. The 10MWT [32] has been found to be valid and reliable in measuring speed in people with PD and will be used as a measure of gait speed during the different time-points in the study. The TUG [33,35,36] has been used as a tool for measuring basic mobility performance and has been proven to be both valid and reliable in people with PD [35]. The MFES [34], unlike the Falls Efficacy Scale (FES) [34,37], assesses outdoor activities as well as indoor activities and demonstrates greater internal test-retest reliability then the original FES. The MFSE will be used to evaluate any change which may occur in an individuals falling pattern due to auditory cueing.

In addition, the impact of the AC device on the individual and their quality of life with be explored using a semi-structured interview. This interview will be conducted by a person unknown to the participant. All interviews will be audio-recorded.

### Participant Selection

The potential participants involved in this study will be volunteers recruited from the Parkinson's Association of Ireland, as well as suitable medically stable candidates recommended by a consultant and/or physiotherapists from a number of hospitals and day centres in Ireland.

### Inclusion/Exclusion criteria

Individuals will be admitted to the study if they present with a formal diagnosis of PD and are recommended for participation to the study by a consultant and/or physiotherapist. Participants on a stable medication regime who freeze at least once a week (minimum score of 2 on item 3 of the FOGQ) and who freeze for at least 2 seconds (minimum score of 1 on item 4 of FOGQ).

Participants will be excluded if they are attending physiotherapy at the time of recruitment. Such participants may demonstrate an improvement in gait pattern following physiotherapy intervention, which may not be attributable to the AC device. Individuals who are unable to use the IACD, who cannot hear the auditory cues and anyone unwilling to provide written informed consent will be excluded. Additionally individuals with acute co-morbidities influencing mobility will also be excluded. Potential participants with a cognitive impairment (< 24 MMSE) or with long/unpredictable "off" periods making stable testing difficult will also be excluded.

### Recruitment

The nominated physiotherapist in each participating centre will be responsible for assessing eligibility for inclusion into the study and also for obtaining informed consent from the patient. Following identification of suitable participants the aims of the project, including the role of the participant will be outlined and any questions answered by the physiotherapist. The person with PD will receive an information brochure in advance of being asked to give written informed consent. Participants will have seven days between receipt of the information brochure and being requested to give written consent. Following the 7-day interval, the physiotherapist will answer or clarify any further questions that arise and the participant will be requested to sign a consent form. If they are unwilling to give written consent, they will be excluded from the study. If they decide to partake in the study, participants will be advised that they can withdraw from the study at any time and it will not interfere with their routine rehabilitation programme. Participants will be allocated a reference code. Names and other details that may identify the participants will be removed.

### Randomisation

To minimise the possibility of recruitment bias, a person independent of the recruitment process will complete random group allocation. Computer generated random numbers will be kept in pre-sealed envelopes in a locked drawer in the Department of Physiotherapy. Allocation will be revealed after recruitment by a telephone call from the physiotherapist to an independent person, who will open the next envelope in the sequence and give the randomisation information to the physiotherapist. Each envelope will only be opened on enrolment of an eligible participant. After allocation has been revealed, the appropriate intervention will be organised by the physiotherapists carrying out the study.

### Power

Sample size is based on power analysis. A sample size calculation was performed for the two quantitative measures, namely the TUG and 10MWT. Power calculations indicate that a total of 47 participants are needed in order to detect with 80% power a difference of 20% between the groups at significance of level of 5%. It is anticipated that it will take 6 months to recruit the required number of participants.

### Analysis

All data will be collected on paper and the records will be stored by registration number in a secure cabinet. Anonymised data will be transferred to a computer database and secured using a password. An independent researcher will cross check all entries. Appropriate statistical tests will be carried out on the data using MINITAB Release 13.1.

The purpose of the statistical analysis is to test the hypothesis that there will be a statistically significant difference in freezing, gait speed and number of falls from baseline to post-intervention assessment. Firstly, the participants' demographic details and primary and secondary outcome measure scores will be compared in the two groups at baseline. If necessary, adjustments for baseline variables will be made using analysis of covariance. Analyses will be carried out to examine the difference between the groups with respect to the change in the FOGQ score from baseline to post-intervention assessment. Secondary outcomes will be compared between the two groups using Student's t-test or the Wilcoxon rank sum test for ordinal data. Descriptive statistics will also be used to represent demographic data.

All interviews will be transcribed verbatim and examined in terms of themes according to the method devised by Miles and Hubberman [39]. Firstly, participants responses to each question will be transferred into a Microsoft excel document to facilitate analysis. Following analysis of the responses to each question, a coding system will be developed in order to facilitate the identification of recurrent responses. Three researchers will be provided with the responses to all of the questions in an unencoded format; thereafter they will independently code the responses sequentially using the predefined codes. Any coding disagreements will be resolved through discussion.

## Discussion

Auditory cueing as a physiotherapy intervention is not a new concept; however, the technology to date has not been practically and commercially viable for use in a clinical setting. By lowering the cueing frequency by 10% of the individuals self-selected walking speed, evidence suggests that this may help to normalise Parkinsonian gait patterns in people who freeze.

The iPod and similar devices provide a cost effective and an innovative platform for integration of IACDs into the clinical setting, social and home environments and are shown to have immediate effect on gait, with improvements in walking speed, stride length and cadence. It is evident that IACDs are of benefit to people with PD and the aim of this RCT is to maximise the benefits by allowing the individual to use devices in both a clinical and social setting, with minimal disruption to their daily routine.

## Conclusion

The self-report questionnaires will allow researchers deeper insight into the impact of IACDs on patients. By establishing evidence based interventions such as described in this protocol, can we strive to reduce the impact of PD related motor symptoms on the individual.

## Competing interests

The authors declare that they have no competing interests.

## Authors' contributions

All authors contributed to the development and writing of the protocol. All authors have been involved in the drafting and revision of this manuscript and have given approval of the final manuscript.

## Pre-publication history

The pre-publication history for this paper can be accessed here:

http://www.biomedcentral.com/1471-2377/8/46/prepub
